# 2-(4-Bromo­benz­yl)-5,11,17,23-tetra-*tert*-butyl-25,26,27,28-tetra­methoxy­calix[4]arene

**DOI:** 10.1107/S1600536809023137

**Published:** 2009-06-27

**Authors:** Conrad Fischer, Guisheng Lin, Wilhelm Seichter, Edwin Weber

**Affiliations:** a Institut für Organische Chemie, TU Bergakademie Freiberg, Leipziger Strasse 29, D-09596 Freiberg/Sachsen, Germany

## Abstract

In the title compound, C_55_H_69_BrO_4_, the calixarene mol­ecule displays a ‘partial cone’ conformation bearing the lateral substituent in a sterically favorable equatorial arrangement between two *syn*-orientated arene units. The crystal packing is stabilized by weak C—H⋯π contacts, involving one *tert*-butyl group, and π–stacking inter­actions of the lateral bromo­benzene units [centroid–centroid distance = 3.706 (1) Å].

## Related literature

For the solid-state structures of laterally monosubstituted calixarenes, see: Biali *et al.* (1996 ▶); Bergamaschi *et al.* (1997 ▶). For laterally unsubstituted calixarenes, see: Fischer *et al.* (2007 ▶, 2008 ▶). For the synthesis of the title compound, see: Scully *et al.* (2001 ▶). For details of C—H⋯π type contacts, see: Nishio (2004 ▶).
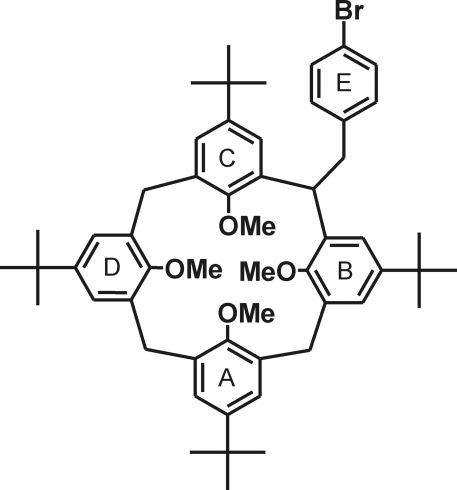


## Experimental

### 

#### Crystal data

C_55_H_69_BrO_4_
*M* *_r_* = 874.01
Triclinic, 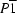

*a* = 10.8146 (5) Å
*b* = 13.8980 (5) Å
*c* = 18.0582 (8) Å
α = 86.473 (2)°
β = 80.442 (3)°
γ = 67.171 (2)°
*V* = 2466.82 (18) Å^3^
*Z* = 2
Mo *K*α radiation
μ = 0.88 mm^−1^
*T* = 153 K
0.38 × 0.18 × 0.10 mm

#### Data collection

Bruker APEXII CCD area-detector diffractometer
Absorption correction: multi-scan (*SADABS*; Sheldrick, 2004 ▶) *T*_min_ = 0.732, *T*_max_ = 0.918
42636 measured reflections
11033 independent reflections
7504 reflections with *I* > 2σ(*I*)
*R*_int_ = 0.036

#### Refinement

*R*[*F* ^2^ > 2σ(*F* ^2^)] = 0.044
*wR*(*F* ^2^) = 0.138
*S* = 1.05
11033 reflections
557 parameters
H-atom parameters constrained
Δρ_max_ = 0.38 e Å^−3^
Δρ_min_ = −0.38 e Å^−3^

### 

Data collection: *APEX2* (Bruker, 2004 ▶); cell refinement: *SAINT* (Bruker, 2004 ▶); data reduction: *SAINT*; program(s) used to solve structure: *SHELXS97* (Sheldrick, 2008 ▶); program(s) used to refine structure: *SHELXL97* (Sheldrick, 2008 ▶); molecular graphics: *ORTEP-3* (Farrugia, 1997 ▶); software used to prepare material for publication: *SHELXTL* (Sheldrick, 2008 ▶).

## Supplementary Material

Crystal structure: contains datablocks global, I. DOI: 10.1107/S1600536809023137/su2121sup1.cif

Structure factors: contains datablocks I. DOI: 10.1107/S1600536809023137/su2121Isup2.hkl

Additional supplementary materials:  crystallographic information; 3D view; checkCIF report

## Figures and Tables

**Table 1:** Hydrogen-bond geometry (Å, °)

*D*—H⋯*A*	*D*—H	H⋯*A*	*D*⋯*A*	*D*—H⋯*A*
C40—H40*A*⋯*Cg*(A)^i^	0.98	2.99	3.8647 (3)	149
C43—H43*C*⋯*Cg*(E)	0.98	2.55	3.5333 (3)	177
C55—H55⋯*Cg*(B)^ii^	0.95	2.98	3.8915 (2)	161

Symmetry codes: (i) 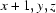; (ii) 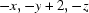. *Cg*(*A*), *Cg*(*B*) and *Cg*(*E*) are the centroids of the C1–C6, C9–C14 and C50–C55 rings, respectively.

**Fig. 1.**
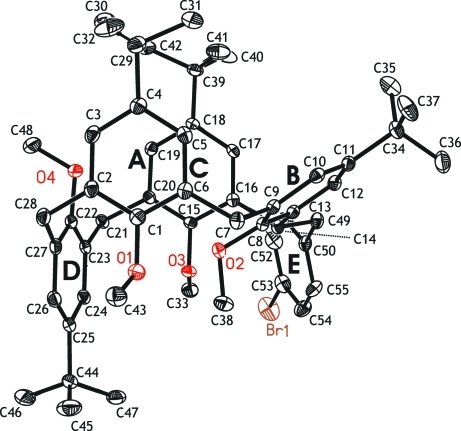
The molecular structure of the title compound, with displacement ellipsoids drawn at the 50% probability level.

**Fig. 2.**
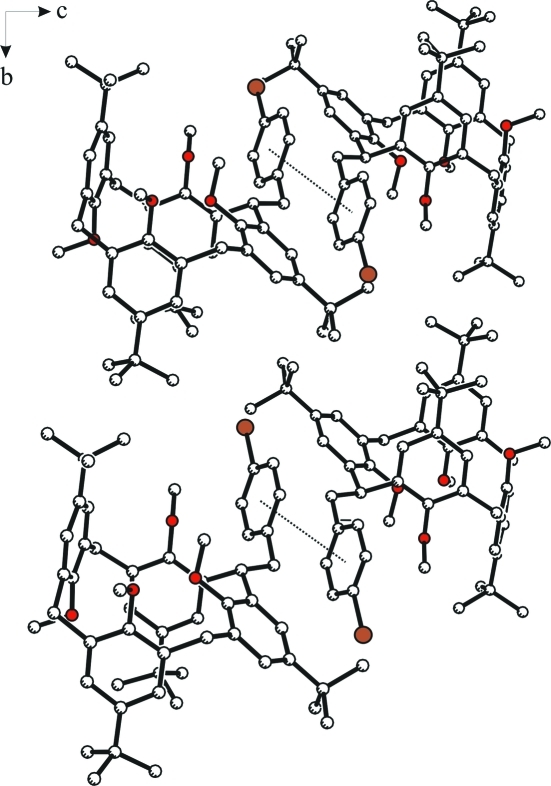
A view down the a axis of the crystal packing of the title compound, illustrating the "handshake" arrangement of opposing bromobenzyl units [Cg(E)···Cg(E)iii = 3.706 (1) Å [symmetry operation: (iii) 1-x,2-y,z].

## supplementary crystallographic information

### Comment

In contrast to the upper and lower rim modified calixarenes, reports on the solid state structures of laterally monosubstituted calixarenes are very rare, including only a calix[4]- and a calix[5]arene with a laterally substituted ethyl group (Biali *et al.*, 1996), and a p-nitrophenyl group (Bergamaschi *et al.*, 1997), in unsolvated and toluene solvated forms, respectively.

The title calixarene (Fig. 1) adopts a *partial cone* conformation, similar to the laterally unsubstituted tetramethoxycalix[4]arene (Fischer *et al.*, 2007, 2008). The opposite arene rings, A (C1-C6) and C (C15-C20), are almost coplanar [dihedral angle 2.31 (1)°], whereas the aromatic rings B (C9-C14) and D (C22-C28) include a dihedral angle of 54.42 (1)°. The lateral *p*-bromobenzyl group [ring E = C50-C55] is located between *syn*-orientated anisole units and its arene unit points to the lower half-space of the calix[4]arene cavity [mpla/E = 74.95 (1)°].

Considering the geometric parameters (Table 1), a C—H···π type contact (Nishio, 2004) between a methoxy group and the lateral arene units [*d*(C43—H43C···Cg(E) = 2.55 (1) Å] is also a feature of the molecular structure. Due to the apolar character of the calixarene habit, the crystal packing behaviour is governed only by weak intermolecular C—H···π contacts involving  *tert*-butyl groups [C—H···Cg(A)^i^ and C-H···Cg(B)^ii^ of 2.99 and 2.98 Å, respectively, and an antiparallel *offset-face-to-face* arrangement of neighboring lateral *p*-bromobenzyl units, Fig. 2 [Cg(E)···Cg(E)^iii^ = 3.706 (1) Å [symmetry operation: (iii) 1-x,2-y,z].

### Experimental

The title calixarene was sythesized from 5,11,17,23-tetra-*tert*-butyl-25,26,27,28-tetramethoxycalix[4]arene following the literature procedure (Scully *et al.*, 2001). Colourless prisms of the solvated calixarene suitable for X-ray diffraction were obtained by recrystallization from ethanol/dichloromethane (m.p. 517–519 K).

### Refinement

The H atoms were positioned geometrically and allowed to ride on their parent atoms, with C—H = 0.95 Å, and *U*_iso_=1.2–1.5 *U*_eq_ (parent atom).

### Crystal data

**Table:**

C_55_H_69_BrO_4_	*Z* = 2
*M**_r_* = 874.01	*F*(000) = 932
Triclinic, *P*1	*D*_x_ = 1.177 Mg m^−^^3^
Hall symbol: -P 1	Mo *K*α radiation, λ = 0.71073 Å
*a* = 10.8146 (5) Å	Cell parameters from 9869 reflections
*b* = 13.8980 (5) Å	θ = 2.3–26.8°
*c* = 18.0582 (8) Å	µ = 0.88 mm^−^^1^
α = 86.473 (2)°	*T* = 153 K
β = 80.442 (3)°	Irregular, colourless
γ = 67.171 (2)°	0.38 × 0.18 × 0.10 mm
*V* = 2466.82 (18) Å^3^	

### Data collection

**Table:**

Bruker APEXII CCD area-detector diffractometer	11033 independent reflections
Radiation source: fine-focus sealed tube	7504 reflections with *I* > 2σ(*I*)
graphite	*R*_int_ = 0.036
φ and ω scans	θ_max_ = 27.3°, θ_min_ = 1.1°
Absorption correction: multi-scan (*SADABS*; Sheldrick, 2004)	*h* = −13→13
*T*_min_ = 0.732, *T*_max_ = 0.918	*k* = −17→17
42636 measured reflections	*l* = −23→23

### Refinement

**Table:**

Refinement on *F*^2^	Primary atom site location: structure-invariant direct methods
Least-squares matrix: full	Secondary atom site location: difference Fourier map
*R*[*F*^2^ > 2σ(*F*^2^)] = 0.044	Hydrogen site location: inferred from neighbouring sites
*wR*(*F*^2^) = 0.138	H-atom parameters constrained
*S* = 1.05	*w* = 1/[σ^2^(*F*_o_^2^) + (0.0738*P*)^2^ + 0.3917*P*] where *P* = (*F*_o_^2^ + 2*F*_c_^2^)/3
11033 reflections	(Δ/σ)_max_ < 0.001
557 parameters	Δρ_max_ = 0.38 e Å^−^^3^
0 restraints	Δρ_min_ = −0.38 e Å^−^^3^

### Special details

**Table:**

Geometry. All e.s.d.'s (except the e.s.d. in the dihedral angle between two l.s. planes) are estimated using the full covariance matrix. The cell e.s.d.'s are taken into account individually in the estimation of e.s.d.'s in distances, angles and torsion angles; correlations between e.s.d.'s in cell parameters are only used when they are defined by crystal symmetry. An approximate (isotropic) treatment of cell e.s.d.'s is used for estimating e.s.d.'s involving l.s. planes.
Refinement. Refinement of *F*^2^ against ALL reflections. The weighted *R*-factor *wR* and goodness of fit *S* are based on *F*^2^, conventional *R*-factors *R* are based on *F*, with *F* set to zero for negative *F*^2^. The threshold expression of *F*^2^ > σ(*F*^2^) is used only for calculating *R*-factors(gt) *etc*. and is not relevant to the choice of reflections for refinement. *R*-factors based on *F*^2^ are statistically about twice as large as those based on *F*, and *R*- factors based on ALL data will be even larger.

### Fractional atomic coordinates and isotropic or equivalent isotropic displacement parameters (Å^2^)

**Table:**

	*x*	*y*	*z*	*U*_iso_*/*U*_eq_	
Br1	0.44092 (3)	1.28131 (2)	0.12208 (2)	0.06814 (13)	
O1	−0.35278 (14)	0.89758 (10)	0.28331 (8)	0.0297 (3)	
O2	−0.08538 (13)	0.90990 (10)	0.18368 (8)	0.0264 (3)	
O3	0.05169 (13)	1.04520 (10)	0.24052 (8)	0.0264 (3)	
O4	−0.01630 (14)	0.80642 (10)	0.42058 (8)	0.0297 (3)	
C1	−0.2624 (2)	0.79587 (15)	0.29288 (12)	0.0264 (4)	
C2	−0.2379 (2)	0.75995 (15)	0.36517 (11)	0.0276 (5)	
C3	−0.1549 (2)	0.65632 (16)	0.37352 (12)	0.0304 (5)	
H3	−0.1417	0.6304	0.4227	0.036*	
C4	−0.0900 (2)	0.58839 (15)	0.31284 (12)	0.0280 (5)	
C5	−0.1081 (2)	0.62960 (15)	0.24190 (12)	0.0268 (4)	
H5	−0.0607	0.5862	0.1994	0.032*	
C6	−0.19397 (19)	0.73297 (15)	0.23057 (11)	0.0247 (4)	
C7	−0.2086 (2)	0.77391 (16)	0.15174 (11)	0.0264 (4)	
H7A	−0.2766	0.8465	0.1541	0.032*	
H7B	−0.2424	0.7311	0.1252	0.032*	
C8	−0.02051 (19)	0.84202 (14)	0.12375 (11)	0.0228 (4)	
C9	−0.07563 (19)	0.77183 (14)	0.10771 (11)	0.0230 (4)	
C10	−0.0041 (2)	0.69790 (15)	0.05109 (11)	0.0246 (4)	
H10	−0.0402	0.6489	0.0403	0.030*	
C11	0.1185 (2)	0.69391 (15)	0.01003 (11)	0.0245 (4)	
C12	0.1693 (2)	0.76668 (15)	0.02775 (11)	0.0254 (4)	
H12	0.2526	0.7654	0.0000	0.031*	
C13	0.10306 (19)	0.84048 (14)	0.08416 (11)	0.0227 (4)	
C14	0.16478 (19)	0.91329 (15)	0.10853 (11)	0.0225 (4)	
H14	0.0923	0.9851	0.1136	0.027*	
C15	0.14395 (18)	0.94370 (14)	0.24908 (11)	0.0213 (4)	
C16	0.20432 (18)	0.87936 (14)	0.18595 (11)	0.0215 (4)	
C17	0.29298 (19)	0.77773 (14)	0.19654 (11)	0.0228 (4)	
H17	0.3360	0.7338	0.1538	0.027*	
C18	0.32171 (19)	0.73727 (14)	0.26690 (11)	0.0232 (4)	
C19	0.25546 (19)	0.80312 (15)	0.32831 (11)	0.0245 (4)	
H19	0.2721	0.7772	0.3771	0.029*	
C20	0.16574 (19)	0.90559 (14)	0.32087 (11)	0.0222 (4)	
C21	0.0853 (2)	0.96957 (15)	0.39057 (12)	0.0259 (4)	
H21A	0.1227	0.9324	0.4354	0.031*	
H21B	0.0957	1.0374	0.3875	0.031*	
C22	−0.1102 (2)	0.90741 (14)	0.41575 (11)	0.0251 (4)	
C23	−0.0644 (2)	0.98905 (15)	0.39987 (11)	0.0244 (4)	
C24	−0.1606 (2)	1.08834 (15)	0.39035 (11)	0.0260 (4)	
H24	−0.1308	1.1444	0.3798	0.031*	
C25	−0.2984 (2)	1.10931 (15)	0.39563 (11)	0.0275 (4)	
C26	−0.3390 (2)	1.02602 (16)	0.41233 (12)	0.0293 (5)	
H26	−0.4329	1.0385	0.4177	0.035*	
C27	−0.2466 (2)	0.92485 (15)	0.42140 (11)	0.0274 (4)	
C28	−0.2937 (2)	0.83421 (16)	0.43159 (12)	0.0309 (5)	
H28A	−0.2643	0.7953	0.4775	0.037*	
H28B	−0.3942	0.8621	0.4387	0.037*	
C29	−0.0067 (2)	0.47202 (16)	0.32560 (13)	0.0344 (5)	
C30	0.0861 (3)	0.4585 (2)	0.38402 (16)	0.0489 (6)	
H30A	0.0308	0.4868	0.4323	0.073*	
H30B	0.1462	0.4960	0.3676	0.073*	
H30C	0.1408	0.3842	0.3896	0.073*	
C31	0.0824 (3)	0.41699 (19)	0.25375 (16)	0.0542 (7)	
H31A	0.1425	0.4529	0.2332	0.081*	
H31B	0.0247	0.4183	0.2169	0.081*	
H31C	0.1370	0.3444	0.2651	0.081*	
C32	−0.1068 (3)	0.4197 (2)	0.35385 (19)	0.0562 (8)	
H32A	−0.0564	0.3460	0.3644	0.084*	
H32B	−0.1631	0.4250	0.3153	0.084*	
H32C	−0.1649	0.4547	0.3999	0.084*	
C33	−0.4914 (2)	0.90741 (19)	0.29668 (15)	0.0407 (6)	
H33A	−0.5068	0.8683	0.2582	0.061*	
H33B	−0.5512	0.9812	0.2946	0.061*	
H33C	−0.5111	0.8794	0.3464	0.061*	
C34	0.1990 (2)	0.61421 (16)	−0.05279 (12)	0.0312 (5)	
C35	0.3405 (2)	0.55112 (19)	−0.03496 (16)	0.0491 (7)	
H35A	0.3340	0.5130	0.0121	0.074*	
H35B	0.3864	0.5982	−0.0296	0.074*	
H35C	0.3924	0.5014	−0.0758	0.074*	
C36	0.2088 (3)	0.6734 (2)	−0.12702 (14)	0.0509 (7)	
H36A	0.2608	0.6233	−0.1676	0.076*	
H36B	0.2544	0.7208	−0.1222	0.076*	
H36C	0.1173	0.7137	−0.1385	0.076*	
C37	0.1311 (3)	0.5392 (2)	−0.06337 (16)	0.0519 (7)	
H37A	0.1266	0.4986	−0.0174	0.078*	
H37B	0.1841	0.4917	−0.1053	0.078*	
H37C	0.0391	0.5790	−0.0743	0.078*	
C38	−0.1770 (2)	1.01055 (17)	0.16531 (14)	0.0377 (5)	
H38A	−0.1257	1.0494	0.1374	0.057*	
H38B	−0.2293	1.0484	0.2116	0.057*	
H38C	−0.2391	1.0031	0.1344	0.057*	
C39	0.4172 (2)	0.62293 (15)	0.27498 (13)	0.0313 (5)	
C40	0.5597 (2)	0.60929 (19)	0.23774 (18)	0.0529 (7)	
H40A	0.5926	0.6520	0.2641	0.079*	
H40B	0.5582	0.6313	0.1852	0.079*	
H40C	0.6203	0.5357	0.2399	0.079*	
C41	0.3683 (3)	0.55236 (18)	0.23535 (17)	0.0502 (7)	
H41A	0.4254	0.4790	0.2428	0.075*	
H41B	0.3746	0.5680	0.1815	0.075*	
H41C	0.2738	0.5651	0.2565	0.075*	
C42	0.4209 (4)	0.5901 (2)	0.35672 (16)	0.0703 (10)	
H42A	0.4803	0.5163	0.3593	0.105*	
H42B	0.3290	0.6003	0.3816	0.105*	
H42C	0.4558	0.6325	0.3819	0.105*	
C43	0.1068 (2)	1.12353 (16)	0.24197 (14)	0.0362 (5)	
H43A	0.1495	1.1147	0.2870	0.054*	
H43B	0.0337	1.1929	0.2427	0.054*	
H43C	0.1747	1.1166	0.1972	0.054*	
C44	−0.3990 (2)	1.22046 (16)	0.38243 (13)	0.0353 (5)	
C45	−0.5324 (3)	1.2205 (2)	0.36486 (18)	0.0580 (8)	
H45A	−0.5755	1.1916	0.4076	0.087*	
H45B	−0.5142	1.1778	0.3201	0.087*	
H45C	−0.5932	1.2922	0.3557	0.087*	
C46	−0.4281 (3)	1.2847 (2)	0.45302 (17)	0.0673 (9)	
H46A	−0.3454	1.2934	0.4606	0.101*	
H46B	−0.4580	1.2488	0.4964	0.101*	
H46C	−0.4996	1.3534	0.4476	0.101*	
C47	−0.3389 (3)	1.26953 (18)	0.31540 (15)	0.0459 (6)	
H47A	−0.4057	1.3387	0.3055	0.069*	
H47B	−0.3152	1.2250	0.2711	0.069*	
H47C	−0.2571	1.2764	0.3266	0.069*	
C48	0.0093 (3)	0.77208 (19)	0.49379 (14)	0.0442 (6)	
H48A	0.0553	0.8115	0.5130	0.066*	
H48B	0.0673	0.6976	0.4924	0.066*	
H48C	−0.0768	0.7833	0.5267	0.066*	
C49	0.2842 (2)	0.91834 (16)	0.05032 (12)	0.0291 (5)	
H49A	0.3630	0.8512	0.0503	0.035*	
H49B	0.2576	0.9282	−0.0003	0.035*	
C50	0.3253 (2)	1.00565 (16)	0.06652 (11)	0.0276 (5)	
C51	0.4286 (2)	0.98924 (17)	0.10800 (13)	0.0342 (5)	
H51	0.4772	0.9209	0.1250	0.041*	
C52	0.4620 (2)	1.07134 (19)	0.12506 (15)	0.0413 (6)	
H52	0.5321	1.0595	0.1542	0.050*	
C53	0.3932 (2)	1.16945 (18)	0.09962 (14)	0.0402 (6)	
C54	0.2911 (3)	1.18814 (19)	0.05744 (14)	0.0433 (6)	
H54	0.2444	1.2563	0.0396	0.052*	
C55	0.2578 (2)	1.10574 (17)	0.04158 (13)	0.0378 (5)	
H55	0.1870	1.1182	0.0129	0.045*	

### Atomic displacement parameters (Å^2^)

**Table:**

	*U*^11^	*U*^22^	*U*^33^	*U*^12^	*U*^13^	*U*^23^
Br1	0.0753 (2)	0.0640 (2)	0.0850 (3)	−0.05400 (18)	0.00914 (17)	−0.01911 (16)
O1	0.0271 (7)	0.0273 (7)	0.0338 (9)	−0.0110 (6)	−0.0017 (6)	0.0014 (6)
O2	0.0267 (7)	0.0285 (7)	0.0233 (8)	−0.0100 (6)	−0.0012 (6)	−0.0051 (6)
O3	0.0271 (7)	0.0186 (6)	0.0307 (8)	−0.0054 (6)	−0.0048 (6)	−0.0005 (6)
O4	0.0366 (8)	0.0207 (7)	0.0272 (8)	−0.0059 (6)	−0.0051 (6)	0.0002 (6)
C1	0.0270 (10)	0.0264 (10)	0.0293 (12)	−0.0160 (8)	−0.0001 (9)	0.0019 (8)
C2	0.0333 (11)	0.0291 (10)	0.0241 (11)	−0.0184 (9)	0.0010 (9)	0.0014 (8)
C3	0.0404 (12)	0.0311 (11)	0.0245 (11)	−0.0205 (9)	−0.0029 (9)	0.0048 (9)
C4	0.0321 (11)	0.0264 (10)	0.0306 (12)	−0.0173 (9)	−0.0046 (9)	0.0031 (9)
C5	0.0284 (10)	0.0277 (10)	0.0271 (11)	−0.0150 (9)	0.0002 (9)	−0.0044 (8)
C6	0.0249 (10)	0.0293 (10)	0.0252 (11)	−0.0168 (8)	−0.0029 (8)	0.0014 (8)
C7	0.0266 (10)	0.0325 (11)	0.0235 (11)	−0.0149 (9)	−0.0040 (8)	−0.0004 (8)
C8	0.0256 (10)	0.0234 (9)	0.0183 (10)	−0.0081 (8)	−0.0041 (8)	0.0004 (8)
C9	0.0256 (10)	0.0256 (10)	0.0193 (10)	−0.0109 (8)	−0.0057 (8)	0.0027 (8)
C10	0.0288 (10)	0.0246 (10)	0.0245 (11)	−0.0130 (8)	−0.0093 (8)	0.0029 (8)
C11	0.0280 (10)	0.0260 (10)	0.0203 (11)	−0.0111 (8)	−0.0040 (8)	0.0001 (8)
C12	0.0270 (10)	0.0283 (10)	0.0220 (11)	−0.0127 (8)	−0.0017 (8)	0.0012 (8)
C13	0.0268 (10)	0.0231 (9)	0.0197 (10)	−0.0109 (8)	−0.0056 (8)	0.0024 (8)
C14	0.0247 (10)	0.0231 (9)	0.0212 (10)	−0.0114 (8)	−0.0016 (8)	−0.0005 (8)
C15	0.0203 (9)	0.0180 (9)	0.0253 (11)	−0.0072 (7)	−0.0028 (8)	−0.0012 (8)
C16	0.0215 (9)	0.0236 (9)	0.0228 (10)	−0.0129 (8)	−0.0023 (8)	−0.0003 (8)
C17	0.0225 (10)	0.0217 (9)	0.0236 (11)	−0.0090 (8)	0.0012 (8)	−0.0056 (8)
C18	0.0209 (9)	0.0198 (9)	0.0272 (11)	−0.0061 (8)	−0.0017 (8)	−0.0027 (8)
C19	0.0250 (10)	0.0272 (10)	0.0221 (11)	−0.0104 (8)	−0.0048 (8)	0.0012 (8)
C20	0.0213 (9)	0.0236 (9)	0.0223 (11)	−0.0090 (8)	−0.0029 (8)	−0.0029 (8)
C21	0.0274 (10)	0.0245 (10)	0.0240 (11)	−0.0080 (8)	−0.0018 (8)	−0.0060 (8)
C22	0.0322 (11)	0.0214 (9)	0.0180 (10)	−0.0069 (8)	−0.0015 (8)	−0.0030 (8)
C23	0.0293 (10)	0.0245 (10)	0.0167 (10)	−0.0076 (8)	−0.0007 (8)	−0.0052 (8)
C24	0.0318 (11)	0.0226 (9)	0.0224 (11)	−0.0103 (8)	0.0002 (8)	−0.0040 (8)
C25	0.0298 (11)	0.0257 (10)	0.0214 (11)	−0.0060 (8)	0.0007 (8)	−0.0026 (8)
C26	0.0270 (11)	0.0326 (11)	0.0256 (11)	−0.0102 (9)	0.0014 (9)	−0.0025 (9)
C27	0.0364 (12)	0.0278 (10)	0.0170 (10)	−0.0131 (9)	0.0018 (9)	−0.0027 (8)
C28	0.0371 (12)	0.0321 (11)	0.0245 (11)	−0.0170 (9)	0.0017 (9)	0.0008 (9)
C29	0.0405 (13)	0.0294 (11)	0.0356 (13)	−0.0151 (10)	−0.0099 (10)	0.0039 (9)
C30	0.0485 (15)	0.0430 (14)	0.0504 (17)	−0.0086 (12)	−0.0166 (13)	−0.0004 (12)
C31	0.0715 (19)	0.0286 (12)	0.0501 (17)	−0.0062 (12)	−0.0067 (14)	−0.0049 (11)
C32	0.0552 (16)	0.0337 (13)	0.087 (2)	−0.0239 (12)	−0.0229 (15)	0.0244 (13)
C33	0.0286 (12)	0.0451 (13)	0.0479 (15)	−0.0141 (10)	−0.0051 (10)	0.0003 (11)
C34	0.0351 (12)	0.0317 (11)	0.0279 (12)	−0.0154 (9)	0.0014 (9)	−0.0078 (9)
C35	0.0431 (14)	0.0411 (14)	0.0577 (18)	−0.0116 (11)	0.0003 (12)	−0.0144 (12)
C36	0.0715 (18)	0.0508 (15)	0.0298 (14)	−0.0254 (14)	0.0018 (12)	−0.0079 (11)
C37	0.0532 (15)	0.0538 (15)	0.0548 (17)	−0.0316 (13)	0.0131 (13)	−0.0281 (13)
C38	0.0274 (11)	0.0320 (11)	0.0481 (15)	−0.0044 (9)	−0.0068 (10)	−0.0037 (10)
C39	0.0329 (11)	0.0200 (10)	0.0355 (13)	−0.0030 (8)	−0.0076 (10)	−0.0026 (9)
C40	0.0312 (13)	0.0311 (12)	0.087 (2)	−0.0005 (10)	−0.0090 (13)	−0.0125 (13)
C41	0.0548 (16)	0.0252 (11)	0.075 (2)	−0.0179 (11)	−0.0151 (14)	−0.0001 (12)
C42	0.104 (3)	0.0325 (14)	0.0407 (17)	0.0122 (15)	−0.0166 (16)	0.0034 (12)
C43	0.0463 (13)	0.0218 (10)	0.0414 (14)	−0.0157 (10)	−0.0023 (11)	−0.0007 (9)
C44	0.0337 (12)	0.0271 (11)	0.0344 (13)	−0.0034 (9)	0.0024 (10)	0.0011 (9)
C45	0.0345 (14)	0.0491 (16)	0.078 (2)	−0.0055 (12)	−0.0102 (14)	0.0203 (15)
C46	0.088 (2)	0.0317 (14)	0.0524 (18)	0.0097 (14)	−0.0074 (16)	−0.0098 (12)
C47	0.0400 (13)	0.0309 (12)	0.0528 (17)	−0.0030 (10)	0.0003 (12)	0.0124 (11)
C48	0.0548 (15)	0.0359 (13)	0.0355 (14)	−0.0082 (11)	−0.0147 (12)	0.0057 (10)
C49	0.0341 (11)	0.0340 (11)	0.0233 (11)	−0.0191 (9)	−0.0009 (9)	0.0007 (9)
C50	0.0277 (10)	0.0336 (11)	0.0239 (11)	−0.0172 (9)	0.0025 (9)	0.0019 (9)
C51	0.0259 (11)	0.0360 (12)	0.0404 (14)	−0.0121 (9)	−0.0037 (10)	−0.0003 (10)
C52	0.0277 (11)	0.0506 (14)	0.0513 (16)	−0.0204 (11)	−0.0052 (11)	−0.0084 (12)
C53	0.0435 (13)	0.0416 (13)	0.0444 (15)	−0.0301 (11)	0.0065 (11)	−0.0060 (11)
C54	0.0565 (15)	0.0364 (13)	0.0428 (15)	−0.0247 (12)	−0.0108 (12)	0.0117 (11)
C55	0.0469 (14)	0.0382 (12)	0.0348 (13)	−0.0216 (11)	−0.0157 (11)	0.0129 (10)

### Geometric parameters (Å, °)

**Table:**

Br1—C53	1.902 (2)	C31—H31A	0.9800
O1—C1	1.392 (2)	C31—H31B	0.9800
O1—C33	1.432 (3)	C31—H31C	0.9800
O2—C8	1.383 (2)	C32—H32A	0.9800
O2—C38	1.423 (2)	C32—H32B	0.9800
O3—C15	1.393 (2)	C32—H32C	0.9800
O3—C43	1.433 (2)	C33—H33A	0.9800
O4—C22	1.385 (2)	C33—H33B	0.9800
O4—C48	1.412 (3)	C33—H33C	0.9800
C1—C6	1.386 (3)	C34—C35	1.517 (3)
C1—C2	1.399 (3)	C34—C37	1.526 (3)
C2—C3	1.385 (3)	C34—C36	1.539 (3)
C2—C28	1.515 (3)	C35—H35A	0.9800
C3—C4	1.394 (3)	C35—H35B	0.9800
C3—H3	0.9500	C35—H35C	0.9800
C4—C5	1.383 (3)	C36—H36A	0.9800
C4—C29	1.540 (3)	C36—H36B	0.9800
C5—C6	1.400 (3)	C36—H36C	0.9800
C5—H5	0.9500	C37—H37A	0.9800
C6—C7	1.510 (3)	C37—H37B	0.9800
C7—C9	1.514 (3)	C37—H37C	0.9800
C7—H7A	0.9900	C38—H38A	0.9800
C7—H7B	0.9900	C38—H38B	0.9800
C8—C9	1.391 (3)	C38—H38C	0.9800
C8—C13	1.400 (3)	C39—C42	1.520 (3)
C9—C10	1.396 (3)	C39—C40	1.520 (3)
C10—C11	1.390 (3)	C39—C41	1.539 (3)
C10—H10	0.9500	C40—H40A	0.9800
C11—C12	1.399 (3)	C40—H40B	0.9800
C11—C34	1.533 (3)	C40—H40C	0.9800
C12—C13	1.385 (3)	C41—H41A	0.9800
C12—H12	0.9500	C41—H41B	0.9800
C13—C14	1.530 (3)	C41—H41C	0.9800
C14—C16	1.523 (3)	C42—H42A	0.9800
C14—C49	1.545 (3)	C42—H42B	0.9800
C14—H14	1.0000	C42—H42C	0.9800
C15—C20	1.392 (3)	C43—H43A	0.9800
C15—C16	1.397 (3)	C43—H43B	0.9800
C16—C17	1.388 (3)	C43—H43C	0.9800
C17—C18	1.391 (3)	C44—C46	1.520 (4)
C17—H17	0.9500	C44—C45	1.527 (4)
C18—C19	1.390 (3)	C44—C47	1.528 (3)
C18—C39	1.536 (3)	C45—H45A	0.9800
C19—C20	1.391 (3)	C45—H45B	0.9800
C19—H19	0.9500	C45—H45C	0.9800
C20—C21	1.519 (3)	C46—H46A	0.9800
C21—C23	1.516 (3)	C46—H46B	0.9800
C21—H21A	0.9900	C46—H46C	0.9800
C21—H21B	0.9900	C47—H47A	0.9800
C22—C27	1.386 (3)	C47—H47B	0.9800
C22—C23	1.401 (3)	C47—H47C	0.9800
C23—C24	1.390 (3)	C48—H48A	0.9800
C24—C25	1.391 (3)	C48—H48B	0.9800
C24—H24	0.9500	C48—H48C	0.9800
C25—C26	1.390 (3)	C49—C50	1.503 (3)
C25—C44	1.537 (3)	C49—H49A	0.9900
C26—C27	1.392 (3)	C49—H49B	0.9900
C26—H26	0.9500	C50—C55	1.384 (3)
C27—C28	1.523 (3)	C50—C51	1.386 (3)
C28—H28A	0.9900	C51—C52	1.388 (3)
C28—H28B	0.9900	C51—H51	0.9500
C29—C31	1.527 (3)	C52—C53	1.369 (3)
C29—C30	1.529 (3)	C52—H52	0.9500
C29—C32	1.530 (3)	C53—C54	1.378 (4)
C30—H30A	0.9800	C54—C55	1.385 (3)
C30—H30B	0.9800	C54—H54	0.9500
C30—H30C	0.9800	C55—H55	0.9500
			
C1—O1—C33	112.62 (16)	H32A—C32—H32C	109.5
C8—O2—C38	115.48 (16)	H32B—C32—H32C	109.5
C15—O3—C43	114.08 (15)	O1—C33—H33A	109.5
C22—O4—C48	115.39 (16)	O1—C33—H33B	109.5
C6—C1—O1	119.65 (18)	H33A—C33—H33B	109.5
C6—C1—C2	120.87 (18)	O1—C33—H33C	109.5
O1—C1—C2	119.43 (18)	H33A—C33—H33C	109.5
C3—C2—C1	118.12 (19)	H33B—C33—H33C	109.5
C3—C2—C28	121.10 (19)	C35—C34—C37	108.6 (2)
C1—C2—C28	120.68 (18)	C35—C34—C11	109.65 (19)
C2—C3—C4	122.8 (2)	C37—C34—C11	112.43 (18)
C2—C3—H3	118.6	C35—C34—C36	109.7 (2)
C4—C3—H3	118.6	C37—C34—C36	107.8 (2)
C5—C4—C3	116.99 (19)	C11—C34—C36	108.64 (18)
C5—C4—C29	122.29 (19)	C34—C35—H35A	109.5
C3—C4—C29	120.67 (19)	C34—C35—H35B	109.5
C4—C5—C6	122.32 (19)	H35A—C35—H35B	109.5
C4—C5—H5	118.8	C34—C35—H35C	109.5
C6—C5—H5	118.8	H35A—C35—H35C	109.5
C1—C6—C5	118.49 (19)	H35B—C35—H35C	109.5
C1—C6—C7	121.59 (18)	C34—C36—H36A	109.5
C5—C6—C7	119.91 (18)	C34—C36—H36B	109.5
C6—C7—C9	112.19 (16)	H36A—C36—H36B	109.5
C6—C7—H7A	109.2	C34—C36—H36C	109.5
C9—C7—H7A	109.2	H36A—C36—H36C	109.5
C6—C7—H7B	109.2	H36B—C36—H36C	109.5
C9—C7—H7B	109.2	C34—C37—H37A	109.5
H7A—C7—H7B	107.9	C34—C37—H37B	109.5
O2—C8—C9	118.68 (17)	H37A—C37—H37B	109.5
O2—C8—C13	119.59 (17)	C34—C37—H37C	109.5
C9—C8—C13	121.58 (17)	H37A—C37—H37C	109.5
C8—C9—C10	118.47 (17)	H37B—C37—H37C	109.5
C8—C9—C7	120.82 (17)	O2—C38—H38A	109.5
C10—C9—C7	120.70 (17)	O2—C38—H38B	109.5
C11—C10—C9	121.91 (18)	H38A—C38—H38B	109.5
C11—C10—H10	119.0	O2—C38—H38C	109.5
C9—C10—H10	119.0	H38A—C38—H38C	109.5
C10—C11—C12	117.54 (18)	H38B—C38—H38C	109.5
C10—C11—C34	123.18 (18)	C42—C39—C40	108.8 (2)
C12—C11—C34	119.28 (17)	C42—C39—C18	112.14 (18)
C13—C12—C11	122.65 (18)	C40—C39—C18	108.85 (18)
C13—C12—H12	118.7	C42—C39—C41	108.8 (2)
C11—C12—H12	118.7	C40—C39—C41	109.2 (2)
C12—C13—C8	117.84 (17)	C18—C39—C41	108.94 (18)
C12—C13—C14	122.67 (17)	C39—C40—H40A	109.5
C8—C13—C14	119.30 (17)	C39—C40—H40B	109.5
C16—C14—C13	108.25 (15)	H40A—C40—H40B	109.5
C16—C14—C49	112.83 (16)	C39—C40—H40C	109.5
C13—C14—C49	112.64 (16)	H40A—C40—H40C	109.5
C16—C14—H14	107.6	H40B—C40—H40C	109.5
C13—C14—H14	107.6	C39—C41—H41A	109.5
C49—C14—H14	107.6	C39—C41—H41B	109.5
C20—C15—O3	119.40 (16)	H41A—C41—H41B	109.5
C20—C15—C16	121.12 (17)	C39—C41—H41C	109.5
O3—C15—C16	119.26 (17)	H41A—C41—H41C	109.5
C17—C16—C15	117.93 (18)	H41B—C41—H41C	109.5
C17—C16—C14	119.54 (17)	C39—C42—H42A	109.5
C15—C16—C14	122.21 (17)	C39—C42—H42B	109.5
C16—C17—C18	122.97 (17)	H42A—C42—H42B	109.5
C16—C17—H17	118.5	C39—C42—H42C	109.5
C18—C17—H17	118.5	H42A—C42—H42C	109.5
C19—C18—C17	116.99 (17)	H42B—C42—H42C	109.5
C19—C18—C39	122.61 (18)	O3—C43—H43A	109.5
C17—C18—C39	120.32 (17)	O3—C43—H43B	109.5
C18—C19—C20	122.39 (19)	H43A—C43—H43B	109.5
C18—C19—H19	118.8	O3—C43—H43C	109.5
C20—C19—H19	118.8	H43A—C43—H43C	109.5
C19—C20—C15	118.50 (17)	H43B—C43—H43C	109.5
C19—C20—C21	119.67 (18)	C46—C44—C45	109.0 (2)
C15—C20—C21	121.61 (17)	C46—C44—C47	110.4 (2)
C23—C21—C20	112.44 (16)	C45—C44—C47	107.3 (2)
C23—C21—H21A	109.1	C46—C44—C25	108.52 (19)
C20—C21—H21A	109.1	C45—C44—C25	111.65 (19)
C23—C21—H21B	109.1	C47—C44—C25	110.01 (17)
C20—C21—H21B	109.1	C44—C45—H45A	109.5
H21A—C21—H21B	107.8	C44—C45—H45B	109.5
O4—C22—C27	119.43 (17)	H45A—C45—H45B	109.5
O4—C22—C23	119.22 (18)	C44—C45—H45C	109.5
C27—C22—C23	121.20 (18)	H45A—C45—H45C	109.5
C24—C23—C22	117.74 (18)	H45B—C45—H45C	109.5
C24—C23—C21	120.86 (18)	C44—C46—H46A	109.5
C22—C23—C21	121.37 (17)	C44—C46—H46B	109.5
C23—C24—C25	122.93 (19)	H46A—C46—H46B	109.5
C23—C24—H24	118.5	C44—C46—H46C	109.5
C25—C24—H24	118.5	H46A—C46—H46C	109.5
C26—C25—C24	117.18 (18)	H46B—C46—H46C	109.5
C26—C25—C44	122.50 (19)	C44—C47—H47A	109.5
C24—C25—C44	120.32 (18)	C44—C47—H47B	109.5
C25—C26—C27	122.13 (19)	H47A—C47—H47B	109.5
C25—C26—H26	118.9	C44—C47—H47C	109.5
C27—C26—H26	118.9	H47A—C47—H47C	109.5
C22—C27—C26	118.80 (18)	H47B—C47—H47C	109.5
C22—C27—C28	120.74 (18)	O4—C48—H48A	109.5
C26—C27—C28	120.31 (19)	O4—C48—H48B	109.5
C2—C28—C27	112.47 (16)	H48A—C48—H48B	109.5
C2—C28—H28A	109.1	O4—C48—H48C	109.5
C27—C28—H28A	109.1	H48A—C48—H48C	109.5
C2—C28—H28B	109.1	H48B—C48—H48C	109.5
C27—C28—H28B	109.1	C50—C49—C14	112.24 (16)
H28A—C28—H28B	107.8	C50—C49—H49A	109.2
C31—C29—C30	107.7 (2)	C14—C49—H49A	109.2
C31—C29—C32	108.7 (2)	C50—C49—H49B	109.2
C30—C29—C32	109.3 (2)	C14—C49—H49B	109.2
C31—C29—C4	112.36 (19)	H49A—C49—H49B	107.9
C30—C29—C4	110.98 (18)	C55—C50—C51	118.2 (2)
C32—C29—C4	107.73 (18)	C55—C50—C49	120.34 (19)
C29—C30—H30A	109.5	C51—C50—C49	121.44 (19)
C29—C30—H30B	109.5	C50—C51—C52	120.9 (2)
H30A—C30—H30B	109.5	C50—C51—H51	119.6
C29—C30—H30C	109.5	C52—C51—H51	119.6
H30A—C30—H30C	109.5	C53—C52—C51	119.5 (2)
H30B—C30—H30C	109.5	C53—C52—H52	120.3
C29—C31—H31A	109.5	C51—C52—H52	120.3
C29—C31—H31B	109.5	C52—C53—C54	121.1 (2)
H31A—C31—H31B	109.5	C52—C53—Br1	119.31 (19)
C29—C31—H31C	109.5	C54—C53—Br1	119.61 (18)
H31A—C31—H31C	109.5	C53—C54—C55	118.8 (2)
H31B—C31—H31C	109.5	C53—C54—H54	120.6
C29—C32—H32A	109.5	C55—C54—H54	120.6
C29—C32—H32B	109.5	C50—C55—C54	121.5 (2)
H32A—C32—H32B	109.5	C50—C55—H55	119.2
C29—C32—H32C	109.5	C54—C55—H55	119.2
			
C33—O1—C1—C6	100.4 (2)	C16—C15—C20—C21	−170.94 (17)
C33—O1—C1—C2	−82.1 (2)	C19—C20—C21—C23	−109.3 (2)
C6—C1—C2—C3	−7.0 (3)	C15—C20—C21—C23	65.2 (2)
O1—C1—C2—C3	175.55 (17)	C48—O4—C22—C27	−89.3 (2)
C6—C1—C2—C28	169.34 (18)	C48—O4—C22—C23	95.2 (2)
O1—C1—C2—C28	−8.1 (3)	O4—C22—C23—C24	175.71 (17)
C1—C2—C3—C4	3.3 (3)	C27—C22—C23—C24	0.3 (3)
C28—C2—C3—C4	−172.99 (19)	O4—C22—C23—C21	−2.1 (3)
C2—C3—C4—C5	1.9 (3)	C27—C22—C23—C21	−177.53 (18)
C2—C3—C4—C29	−175.78 (19)	C20—C21—C23—C24	−110.4 (2)
C3—C4—C5—C6	−3.8 (3)	C20—C21—C23—C22	67.4 (2)
C29—C4—C5—C6	173.90 (18)	C22—C23—C24—C25	−0.3 (3)
O1—C1—C6—C5	−177.27 (16)	C21—C23—C24—C25	177.61 (19)
C2—C1—C6—C5	5.3 (3)	C23—C24—C25—C26	1.0 (3)
O1—C1—C6—C7	3.7 (3)	C23—C24—C25—C44	−178.38 (19)
C2—C1—C6—C7	−173.79 (17)	C24—C25—C26—C27	−1.8 (3)
C4—C5—C6—C1	0.3 (3)	C44—C25—C26—C27	177.51 (19)
C4—C5—C6—C7	179.34 (18)	O4—C22—C27—C26	−176.52 (18)
C1—C6—C7—C9	117.0 (2)	C23—C22—C27—C26	−1.1 (3)
C5—C6—C7—C9	−62.1 (2)	O4—C22—C27—C28	−1.0 (3)
C38—O2—C8—C9	−95.2 (2)	C23—C22—C27—C28	174.40 (18)
C38—O2—C8—C13	89.2 (2)	C25—C26—C27—C22	1.9 (3)
O2—C8—C9—C10	−174.90 (17)	C25—C26—C27—C28	−173.61 (19)
C13—C8—C9—C10	0.6 (3)	C3—C2—C28—C27	118.7 (2)
O2—C8—C9—C7	3.8 (3)	C1—C2—C28—C27	−57.5 (3)
C13—C8—C9—C7	179.29 (18)	C22—C27—C28—C2	−63.4 (3)
C6—C7—C9—C8	−72.2 (2)	C26—C27—C28—C2	112.1 (2)
C6—C7—C9—C10	106.4 (2)	C5—C4—C29—C31	15.9 (3)
C8—C9—C10—C11	−1.0 (3)	C3—C4—C29—C31	−166.6 (2)
C7—C9—C10—C11	−179.70 (18)	C5—C4—C29—C30	136.5 (2)
C9—C10—C11—C12	0.5 (3)	C3—C4—C29—C30	−45.9 (3)
C9—C10—C11—C34	−179.59 (19)	C5—C4—C29—C32	−103.9 (2)
C10—C11—C12—C13	0.5 (3)	C3—C4—C29—C32	73.7 (3)
C34—C11—C12—C13	−179.44 (19)	C10—C11—C34—C35	−122.3 (2)
C11—C12—C13—C8	−0.9 (3)	C12—C11—C34—C35	57.6 (3)
C11—C12—C13—C14	174.12 (18)	C10—C11—C34—C37	−1.4 (3)
O2—C8—C13—C12	175.79 (17)	C12—C11—C34—C37	178.5 (2)
C9—C8—C13—C12	0.3 (3)	C10—C11—C34—C36	117.9 (2)
O2—C8—C13—C14	0.6 (3)	C12—C11—C34—C36	−62.2 (3)
C9—C8—C13—C14	−174.87 (18)	C19—C18—C39—C42	4.3 (3)
C12—C13—C14—C16	−108.8 (2)	C17—C18—C39—C42	−172.5 (2)
C8—C13—C14—C16	66.1 (2)	C19—C18—C39—C40	−116.2 (2)
C12—C13—C14—C49	16.7 (3)	C17—C18—C39—C40	67.0 (3)
C8—C13—C14—C49	−168.40 (18)	C19—C18—C39—C41	124.8 (2)
C43—O3—C15—C20	83.1 (2)	C17—C18—C39—C41	−52.0 (3)
C43—O3—C15—C16	−102.1 (2)	C26—C25—C44—C46	101.8 (3)
C20—C15—C16—C17	−3.6 (3)	C24—C25—C44—C46	−78.9 (3)
O3—C15—C16—C17	−178.27 (16)	C26—C25—C44—C45	−18.4 (3)
C20—C15—C16—C14	169.83 (17)	C24—C25—C44—C45	160.9 (2)
O3—C15—C16—C14	−4.8 (3)	C26—C25—C44—C47	−137.4 (2)
C13—C14—C16—C17	56.9 (2)	C24—C25—C44—C47	41.9 (3)
C49—C14—C16—C17	−68.4 (2)	C16—C14—C49—C50	−68.9 (2)
C13—C14—C16—C15	−116.40 (19)	C13—C14—C49—C50	168.14 (17)
C49—C14—C16—C15	118.25 (19)	C14—C49—C50—C55	−84.5 (2)
C15—C16—C17—C18	1.3 (3)	C14—C49—C50—C51	94.0 (2)
C14—C16—C17—C18	−172.33 (17)	C55—C50—C51—C52	1.0 (3)
C16—C17—C18—C19	1.0 (3)	C49—C50—C51—C52	−177.5 (2)
C16—C17—C18—C39	177.94 (17)	C50—C51—C52—C53	−1.0 (3)
C17—C18—C19—C20	−1.0 (3)	C51—C52—C53—C54	0.1 (4)
C39—C18—C19—C20	−177.87 (18)	C51—C52—C53—Br1	−179.20 (17)
C18—C19—C20—C15	−1.3 (3)	C52—C53—C54—C55	0.6 (4)
C18—C19—C20—C21	173.39 (18)	Br1—C53—C54—C55	179.95 (18)
O3—C15—C20—C19	178.27 (16)	C51—C50—C55—C54	−0.3 (3)
C16—C15—C20—C19	3.6 (3)	C49—C50—C55—C54	178.2 (2)
O3—C15—C20—C21	3.7 (3)	C53—C54—C55—C50	−0.6 (4)

### Hydrogen-bond geometry (Å, °)

**Table:**

*D*—H···*A*	*D*—H	H···*A*	*D*···*A*	*D*—H···*A*
C40—H40A···Cg(A)^i^	0.98	2.99	3.8647 (3)	149
C43—H43C···Cg(E)	0.98	2.55	3.5333 (3)	177
C55—H55···Cg(B)^ii^	0.95	2.98	3.8915 (2)	161

Symmetry codes: (i) *x*+1, *y*, *z*; (ii) −*x*, −*y*+2, −*z*.
